# Exploring the critical points of teaching STEM subjects in the time of COVID 19: the experience of the course "Microscopy Techniques for Forensic Biology"

**DOI:** 10.12688/f1000research.28455.2

**Published:** 2021-04-12

**Authors:** Elvira Brunelli, Rachele Macirella

**Affiliations:** 1 Deptarment of Biology, Ecology and Earth Science (DiBEST), University of Calabria, Rende, 87036, Italy

**Keywords:** Covid-19 outbreak, online learning, STEM, Science laboratory, Classroom climate.

## Abstract

**Background**: The University was among the first structures to be hit by the health emergency, transferring all its teaching and research activities remotely. It was not easy for teachers and students to find themselves suddenly shifted into different teaching and socializing context.

**Results**: This article describes and analyzes the online teaching experience carried out for the course of Microscopy Techniques for Forensic Biology offered as a part of the Master's degree program in Biology at the University of Calabria (Italy). A cross-sectional survey (pilot study) was designed to investigate the accessibility of distance learning along with an evaluation of adjustments needed for the conversion from offline to online instruction. Particular attention has been paid to learning material and lesson duration, with specific emphasis on practical activities.

**Conclusions**: The author's intent is that of opening a comparison between the strengths and weaknesses that emerged in this experience, highlighting, in particular, how the educational relationship between teacher and student has changed.

## Introduction

The traditional academic and research community asked learners to handle paper-based documents, taking the form of exercise books, notebooks, lecture notes, etc., and a way of transmitting knowledge mainly based on the frontal lessons. The use of modern technologies and e-learning-based culture partially modified the formulas of education ( Singh & Thurman, 2019); the use of digital resources is now very diffuse, also representing a useful communication system for the needs of science. According to the European Commission’s directives, relating to the Lifelong Learning Program, which promotes the transformation of education through technology, ( https://eur-lex.europa.eu/legal-content/EN/TXT/?uri=CELEX%3A52004PC0474), an e-learning service to support didactics ( https://24cfu.unical.it/elearning/) has been created at the University of Calabria, Italy. The overall objective envisaged was the enrichment of traditional lessons through the use of network technologies and related paradigms. This e-learning service relies on Massive Open Online Courses (MOOCs) that support distance education, acknowledging the existence of a plurality of academic training offers ( Kaplan & Haenlein, 2016). Despite these growing innovations, until now, the most common teaching method in university education remains the face-to-face lessons that immediately enable the teacher and the students to communicate easily and confidently.

For preventing the expansion of COVID-19, in Italy, restrictive measures were adopted almost immediately, leading to the closure of higher education institutions and the suspension of face-to-face activities on March 5, 2020. Given the medium to the long-term perspective of suspension, teachers were all asked to deliver their lectures via the Internet to ensure education and give students the continuation of their intellectual experience. To guarantee a standard implementation of courses and facilitate both teachers and students, the University of Calabria found an adequate settlement through the choice of the Microsoft Teams platform (©Microsoft Teams Version 1.3.00.3564 for Windows and 2.0.15 for iOS, Microsoft Corporation). In this scenario, the students experienced a sudden and unwanted time and spatial separation, and the lesson delivery shift was not readily accepted. While the focus is mainly placed on the impacts on students, teachers also faced some challenges, including the choice of instructional strategies, learning resources, and assessment methods. Besides, since from a constructivist perspective enhancing interaction with peers is an issue that should be addressed, it is crucial to pay adequate attention to the interactivity during online learning ( Huang *et al.*, 2019).

Furthermore, the conversion from an offline to online instruction when courses include the development of specific competencies through practice, as is the case in science, technology, engineering, and math (STEM) courses, required special attention. Although STEM education has drawn increased consideration in the last few years, little attention has been paid on how it should be realized in practice during online learning. Here, we report the online teaching experience for a course in the STEM area, offered as a part of the Master’s degree program in biology. The course includes both lessons and practical laboratory sessions delivered for 21 students with a high level of confidence in using digital tools and web-based applications. Despite their digital background, developed in different, non-educational contexts, students were unfamiliar with e-learning tools, and they previously experienced only traditional teaching that featured lessons, laboratory experiences, and assignments delivered in person. This paper aims to participate in the dialogue about online education during the pandemic contributing to the definition of the future strategy aimed to overcome the limits of non-traditional learning.

## Methods

### The research framework

The platform used for delivering online lessons was Microsoft Teams (©Microsoft Teams Version 1.3.00.3564 for Windows and 2.0.15 for iOS, Microsoft Corporation). The software Teams, like several other applications, allows different modes to hold virtual classes i) pre-recorded audio incorporated in the slide presentation files, ii) recorded classes, allowing students to access on-demand; iii) virtual classes, for students to watch in real-time ( Rainbow, 2020). Before beginning their activity, all teachers attended the University’s training, coordinated by the office in charge, to become familiar with the platform and acquire basic knowledge on how to use, create, and share resources from the perspective of open education ( Bussis & Chittenden, 1970). The same office issued a memorandum addressed to teachers and students, giving the information on software functions and explaining the platform’s essential tools.

The course of “Microscopy Techniques for Forensic Biology” is a core course offered as a part of the Master’s degree program in biology. It includes 36 hours of lessons and 24 hours of practical laboratory work, reaching a total of six ECTS credits (according to the European Credit Transfer and Accumulation System). The course began on March 16, 2020, and ended on June 08, 2020, and was attended by 21 students. The course was delivered entirely online, holding a virtual class supported by slides projection and integrated with other online support such as pre-recorded video, particularly for laboratory work. A downloadable version of the course program was available from the beginning of the academic year on the website of the degree program in biology. The program comprised the synthetic content of the course, divided into 13 topics. It also included the list of suggested texts (e-books and paper-books) and several online resources that are useful to address each topic. On the contrary, the learning material (i.e., a printable version of slides) was shared on the platform just before the official beginning of the course. The slides provided, organized according to the 13 topics reported in the program, were numbered ( Table 1). It was, therefore, possible to compare the material produced at the end of the course.

**Table 1.** Slides and videos for 2019/2020 academic year and percentage change compared to 2018/2019.

	Academic year 2018/2019	Academic year 2019/2020	
Topic	n° slides	n° slides	n° videos
Introduction to microscopy and different types of microscopes	13	15 (+15.38%)	-
History of the microscope	21	21	-
The eye as an optical instrument	18	23 (+27.78%)	-
The stereomicroscope	15	21 (+40.00%)	1
Basic principles of image formation	56	68 (+21.43%)	-
The structures of the light microscope and Köhler illumination	24	28 (+16.67%)	2
Samples preparation for light microscopy observation	33	38 (+15.15%)	2
The staining and immunofluorescence techniques	56	63 (+12.50%)	1
The transmission electron microscope (TEM) - image formation	33	38 (+15.15%)	2
Samples preparation for transmission electron microscopy	55	67 (+21.82%)	2
Laser Scanning confocal microscope - Other microscopes	20	25 (+25.00%)	1
The scanning electron microscope (SEM) - image formation	38	38 (+19.05%)	-
Samples preparation for scanning electron microscopy	21	25 (+15.38%)	1
**Total**	403	474 (+17.62%)	12

### Statistical analysis

A cross-sectional survey (pilot study) was designed to investigate the accessibility of distance learning. An online self-administered survey was conducted in June-July 2020. Questionnaires received after July 7, 2020, were excluded from the study. Questionnaires were completed using the online tool Google Forms. Given the aim of the study, the sample size was not *a priori* determined, and no ethics committee approval was required. None of the participants shared personal information, and the data of the students were anonymous. Participation in the survey did not require approval from an ethics committee as the online questionnaire’s completion was on a voluntary basis and anonymous; it was impossible to track the sensitive personal data of all participants (including the IP address). Before starting the questionnaire, a brief description of the study, the declaration of confidentiality and anonymity were furnished to students. Answers were saved by clicking on the button “submit” and the students could suspend the questionnaire at any time.

The online questionnaire has complied with national and international agreements and regulations, with the Declaration of Helsinki (2000) and the General Data Protection Regulation of the European Parliament (GDPR 679/2016). In the invitation email and the introduction to the survey, we explained research purposes and that the University of Calabria was responsible for data collection and management. We specified that the project and its findings were going to be published in scientific articles. The questionnaire was written in Italian and comprised 16 questions (single-choice questions and one open question); an English translation of the questionnaire is provided in Table 2. For seven questions, answers were scored using a Likert-type scale from 1 to 4 ( Likert, 1932). Survey length, questions suitability, and non-ambiguity of the definitions were considered before survey administration.

**Table 2.** Distance learning during lockdown.

N.	Questions	Total Sample (n = 17)	%
**1**	**Did you attend any online lessons during the COVID19 lockdown?**		
	Yes, the lessons took place entirely during the 2nd semester	17	100.0
	Yes, the lessons started before the emergency and continued during the COVID-19 lockdown	0	0.0
	No, the lessons ended before the lockdown	0	0.0
	No, the lessons did not take place/were suspended	0	0.0
	No, I had difficulty accessing distance learning	0	0.0
**2**	**What is the percentage of the lessons you have attended online?**		
	Not attending or less than 50%	0	0.0
	More than 50%	17	100.0
	I attended/took this exam abroad during the Erasmus program	0	0.0
**3**	**With which device have you been able to follow distance lessons mainly?**		
	PC/Tablet for exclusive use	13	76.5
	PC/Tablet shared with other people and/or smartphones	4	23.5
**4**	**With which type of connection did you mainly have access to the Internet to follow** **distance lessons?**		
	Flat-rate fixed network/Mobile network GB unlimited	12	70.6
	Consumer fixed network / GB mobile network limited	5	29.4
**5**	**In which modality has the distance teaching been carried out?**		
	Only synchronous (streaming video lessons, with possible registration available)	17	100.0
	Only asynchronous (audio-video lessons pre-recorded)	0	0.0
	Mixed (synchronous and asynchronous)	0	0.0
**6**	**In your experience, which type of lessons delivery, do you think is most effective?**		
	Recorded audio lessons (also included in slides and/or pdf)	2	11.8
	Recorded audio lessons (asynchronous)	0	0.0
	Streaming lessons	15	88.2
**7**	**What is your experience with distance learning?**		
	I was comfortable, and I am learning as much as with face-to-face lessons	5	29.4
	I learnt a little less, but I am developing other skills	1	5.9
	I learnt enough, but sometimes I felt tired	10	58.8
	I learnt little, but I tried to adapt to the situation	1	5.9
	I learnt very little because I do not like distance learning	0	0.0
**8**	**What is your opinion regarding distance learning?**		
	Teachers should also integrate it regularly in typical lessons	3	17.7
	During regular courses, it can be useful only for particular needs	10	58.8
	It should only be used in emergencies	4	23.5

Data were exported in an Excel file (Microsoft Corp., Redmond, WA, USA) and analyzed by STATA16 (Version 16.1 for Mac, Stata Corp., College Station, TX, USA). Descriptive statistics included mean and standard deviations (SD) or absolute and relative frequencies. We calculated full-scale internal consistency (Cronbach’s alpha coefficient) for seven items. Two independent authors (VC and AM) analyzed free text responses to determine possible, significant uncertainties aspects of distance learning.

## Results

*Learning material and duration of the lessons -* The learning material (i.e., slides) used previously during the frontal lessons required a significant rearrangement for online delivery. Although the program of the course has remained unchanged compared to previous academic years, online learning has needed the introduction of additional slides. It was necessary to include new slides for 11 of the 13 topics as provided for by the program, and the number of slides as a whole increased by 17.62% ( Table 1). Regarding the laboratory activity, online teaching indeed represented a challenge that has been, in part, overcome through two types of approaches. Whenever possible, tutorials were proposed for supporting the realization of experiences at home by using materials readily available. When laboratory experience required the use of specific instrumentation and supplies, videos of the experience/instrumentation have been prepared in the laboratory and then edited by inserting comments and explanations. A total of 12 videos were made and shared on the platform ( Table 1).

Although no exact quantification was made, the online lessons’ duration was always higher than scheduled before. For each lesson, which was meant to last for two hours in the timetable, at least half an hour more was provided; considering that there were no breaks during the online lesson, the increase could be regarded as rather ample.

*The survey -* 18 students responded to the survey, and 17 of them were included. 88.2% (15/17) of students were female, and 11.8% (2/17) were male. All students attended online lessons during the COVID-19 lockdown. During online lessons, 76.5% (13/17) of students used PC/Tablet for exclusive use, while 23.5% (4/17) of students were using devices shared with other people. The primary type of Internet access was through a flat-rate fixed network or mobile network with unlimited gigabytes (70.6%, 12/17). Streaming lessons were perceived as the most effective kind of remote lessons (88.2%, 15/17). Overall, distance learning has been good for 29.41% (5/17) of students; many students (58.8%, 10/17) found the online lessons useful even if more tiring. 58.8% (10/17) thought that online learning could be an excellent method to be used during regular activity, but only for particular situations; 17.7% (3/17) believed that online learning should be used during the conventional activity as an alternative learning method. 23.5% (4/17) thought that distance learning could be used only in case of emergencies. All results were reported in Table 2.

Of students, 58.8% (15/17) thought that the platform used for distance teaching (Teams) had been usable enough, followed by 35.3% (6/17), who believed that the platform had been entirely usable. 52.9% (9/17) of students held distance learning adequate for self-study. The level of interaction with teachers and other students was thoroughly satisfying for 94.1% (16/17) and 64.7% (11/17), respectively. During the COVID-19 lockdown, communication with teachers was indicated as good enough by 94.1% of students (16/17). For what concern the level of friendliness perceived during online lessons: 29.4% (5/17) and 41.2% of the interviewees thought there was an adequate level of friendliness, similar to that achieved during face-to-face lessons, while 17.7% (3/17) and 11.8% (2/17) had not perceived a high level of friendliness during online lessons. For 58.8% (10/17) of students, the pandemic situation affected learning negatively; 29.4% (5/17) and 11.8% (2/17) did not perceive any correlation between the general sense of anxiety and his/her learning approach. The results were reported in Table 3. Nine students highlighted some problematic aspects of distance learning: the lack of good Internet access and human interactions with teachers and colleagues, and the difficulties in case of technical applications (as microscope use) were the most common issues about distance learning. Internal consistency (Cronbach’s alpha) was equal to 0.68. The value alpha was good, given the aim and field of the study.

**Table 3.** Perceptions about distance learning.

Items	Freq	%	Mean	SD
**The platform used for distance teaching (Teams) has been usable.**				
Definitely No	0	0.0	3.3	0.6
Probably No	1	5.9		
Probably Yes	10	58.8		
Definitely	6	35.3		
**Distance teaching helped self-study.**				
Definitely No	0	0.0	3.4	0.7
Probably No	2	11.8		
Probably Yes	6	35.3		
Definitely	9	52.9		
**Online lessons allow us to interact with the teacher.**				
Definitely No	0	0.0	3.9	0.2
Probably No	0	0.0		
Probably Yes	1	5.9		
Definitely	16	94.1		
**Online lessons allow us to interact with other students.**				
Definitely No	0	0.0	3.6	0.6
Probably No	1	5.9		
Probably Yes	5	29.4		
Definitely	11	64.7		
**Communication with the teacher was satisfying during COVID19** **lockdown.**				
Definitely No	0	0.0	3.9	0.2
Probably No	0	0.0		
Probably Yes	1	5.9		
Definitely	16	94.1		
**During online lessons, the atmosphere was more friendly than during** **face-to-face lessons.**				
Definitely No	3	17.7	2.9	1.1
Probably No	2	11.8		
Probably Yes	5	29.4		
Definitely	7	41.2		
**The pandemic anxiety perceived during COVID19 lockdown impacted** **negatively on my learning. (R)**				
Definitely No	5	29.4	2.7	1.0
Probably No	2	11.8		
Probably Yes	9	52.9		
Definitely	1	5.9		

(R) Reverse Item

## Discussion

To allow the teaching activities to be conducted at an appropriate level, the first hurdle to overcome is the availability of technological supplies and minimal skills to both students and teachers that lack their own resources. Therefore, when evaluating the online teaching experience, it is essential to exclude that technological limits (related to the use of the web or digital tools) or other external issues would affect the results. In the case presented here, no relevant problems have been raised regarding the use of online tools during the whole course since, as expected, students demonstrated to be familiar with digital resources. At all events, the use of a digital environment requiring little or no specific equipment is preferable in order to cater to everyone.

Overall, our results revealed that online learning is considered an excellent method to use only for particular situations or in emergencies. It should be noted that virtual teaching was not foreseen as part of the education course they had chosen, while the students’ expectations are different if they decided to enrol in a distance education course from the beginning. Online synchronous lectures have the characteristics of immediacy, low difficulty, and low cost for both teachers and students ( Xie & Zhang, 2020). Accordingly, from our survey, it emerged that they are considered to be the most useful type of online lessons.

Although the platform used for virtual classes (Teams) was evaluated from enough to entirely usable by most of the students, it is important to highlight some aspects that may be relevant. This platform allows us to see simultaneously up to nine participants on the screen during a video call, so even in a class with a low number of students, like that described here, it is impossible to have an overview of all the students. This restriction forces the teacher to switch through the users’ screen during the lesson since the goal is to include, not exclude, any students. In an attempt to get a picture of the whole class, managing the slides/teaching materials may become difficult. Moreover, the interaction is particularly important in the STEM context ( Huang *et al.*, 2020; McDavid *et al.*, 2020) as teachers can target the learning skills and allow students to feel connected to each other, thus building a trustful learning environment. Therefore, online teaching management needs to consider the number of students to avoid such limitations compared to face-to-face lessons that allow us to have a direct, at least visual, contact with all the learners.

Interestingly, the students’ attempts to interact with the teacher posing questions, were positively affected by the online delivery of the lessons. On average, at the end of a lesson, the number of questions asked was 2–3, while during online teaching, the number per lesson was never less than 5–6. One may suppose that this is related to less successful communication due to distance learning. However, the complexity level of the questions raised during this course suggested that students needed feedback from the teacher more than a clarification of scientific contents. Sometimes, the mainstream environment does not provide an appropriate level of confidence to the more reserved students, and their participation in the academic discussion remains limited; these students would benefit from a different level of communication access. They overcome their restraint during online experience thanks to the ability to manage their image in the video call (i.e., shutting down the video when speaking or using a low-resolution video) or use chat for conversations. On the other hand, the most confident students have not lost their relationship skills.

The higher duration of the online lessons observed here may be partly due to the increased number of questions posed by students that took some time to answer exhaustively. On the other hand, the students had more free time in the absence of extra-university activities and the lack of limits deriving from logistics (i.e., dependence on fixed hours for public transport, travel times, etc.). The absence of these limits allowed us to dwell on the most relevant aspects by dedicating attention not conditioned by compliance with the timetable ( Deci & Ryan, 2000).

For what concerns the learning material, from our results emerge an increase in the number of the slides needed for online compared to face-to-face lessons. It seems that classroom interaction plays a fundamental role by allowing the teacher to identify any perplexities or the decline in student attention quickly. It was, therefore, necessary to introduce some slides with the dual objective of bringing students’ attention back to the right level and reinforcing information during online lessons.

From the perspective of the instructional design, managing the instructional process efficiently will ensure effective and retentive learning. The success of the teaching activity in such contexts relies on the teacher’s ability to use multiple technologies and tools to reorganize the teaching and learning process ( Merrill *et al.*, 1996).

However, for all the possibilities that technology brings to education, one major problem remains when facing practical skills learning; to develop a virtual laboratory class for a master’s degree course requires specific skills, both scientific and technological. The role of virtual simulations has been explored first in science and engineering education. More recently, the usefulness of the virtual laboratory in comparison with other instructional methods has been investigated in the life sciences undergraduate courses, however leading to conflicting results ( Bonde *et al.*, 2014; Dyrberg *et al.*, 2017; Hofstein & Lunetta, 2004). The suitability of tools depends on the educational purpose, and a different approach is required for practical activities.

Maybe the academic community will need to become more involved by providing resources, expertise, and guidance aimed at the development of specific software/tools for laboratory teaching. At the moment, online education has revealed all its limits regarding students’ achievement of adequate familiarity with scientific instrumentation, and the research in this area requires further investigations.

An education program that includes face-to-face and online lectures might represent a possible learning scenario in all situations requiring restrictive measures but also as an independent, alternative learning method. This blended way of learning ( Staker & Horn, 2012) may also take advantage of MOOCs ( Kaplan & Haenlein, 2016; Moreno-Marcos *et al.*, 2019) as additional resources based on the learner characteristics, contents, and resources required.

Classical face-to-face teaching should apparently encourage communication. Surprisingly, online lessons have somewhat promoted communication between users of the platform. Maybe, the particular social situation caused by the pandemic and the related emotional aspects must be taken into account, which may have amplified students’ communication needs. On the other hand, it must be emphasized that enrolled students were not accustomed to online teaching, which may have influenced their perception of class climate. They probably recognized remote teaching as more familiar, partly due to the innovative character of the experience. In fact, the classic Italian system does not routinely use e-learning.

The virtual room allows students to identify themselves as members of a cultural and micro-culture group, and not to endorse the classical model, which classifies teachers and students as separate categories. In the same way, this offered the teacher the opportunity to become a part of the students’ group. The unavoidable intrusion into each other’s daily realities helped in developing a relaxed atmosphere in discussions. The natural trust that has been generated has favored the development of a highly empathic class atmosphere by also facilitating the discussion of scientific contents.

It must also be considered that a different teaching and learning culture exists in different countries. Indeed, a reflection on the teacher-learner relationship in Italian education system and how to improve the relational aspects through the use of new technologies is necessary.

These empirical results are in agreement with the research literature on the role of an informal learning environment as a promoter of motivation and contributing factor to the academic success of students in the area of STEM ( McDavid *et al.*, 2020; Salmi & Thuneberg, 2019).

### Limitations of the study and future research

It is important to note two critical limitations of our study. The most relevant relates to the study design and context. This study aimed to point out the outcomes of a sudden switch from traditional face-to-face to online instruction during the COVID-19 outbreak. Still, the research design was not a controlled experiment with an intervention and control group. The other explicit limitation is the number of participants in the survey. However, the present research is proposed as a case study that becomes more prominent when considering the peculiar conditions that occurred during the pandemic. Moreover, although qualitative, the observed results reveal some dynamics of noteworthy relevance regarding online teaching and its future evolution in the STEM area. For this study’s purpose, a theoretical-practical course was considered, thus highlighting the pros and cons of online learning in science and the importance of flexibility across education systems.

The added value of this study over previous ones is that it refines the role of the educational environment in STEM education, disclosing several unexpected outputs.

## Conclusion

Our study’s results highlighted the strengths and weaknesses of online teaching in the STEM area that emerged during the closure of higher education institutions and the suspension of face-to-face lessons due to the pandemic. The main challenges that emerged from our study concern the practical activities that emphasize that online teaching cannot merely be a transposition of face-to-face lessons in a virtual context. To elaborate specific online teaching for practical courses choosing the more suitable tools and enhancing mutual interaction requires special attention. Accurate planning and organization are needed when implementing STEM education, and a major role of the academic community is envisaged as the promoter of advanced, effective tools development for laboratory teaching. More investigation on this topic is necessary to address limits about the student’s scientific achievements during virtual instruction. Furthermore, promising outcomes arose from this study. They were evident in the learners’ motivations and enhanced communication skills leading to reconsider by the way the educational relationship between teacher and student. In our opinion, when applicable, the blended learning would be the more suitable education program for STEM courses.

## Data availability

### Underlying data

Figshare: Questions in English and Italian, https://doi.org/10.6084/m9.figshare.13656029 ( Brunelli & Macirella, 2021).

Data are available under the terms of the Creative Commons Zero “No rights reserved” data waiver (CC0 1.0 Public domain dedication).

## Consent

Before starting the survey all student have been informed of methods and goals of this work. In the email along with the link to accede the survey students have been provided with following information:

“Participation in the online questionnaire”

This questionnaire is aimed exclusively at the collection of data and information for the evaluation of online teaching. It does not in any way replace the questionnaire that the University submits to students of the final assessment of the course. The data collected will be used exclusively for the preparation of scientific work.

Taking part in the survey implies the declaration of consent. Before completing the questionnaire, please carefully read the information below:

1. the online questionnaire’s completion is on a voluntary basis and anonymous; the questionnaire has been created using the online tool Google Forms. More information on how Google treats data can be found at this link: https://policies.google.com/privacy/update. These data will not be recorded on other media or devices, nor will any other data deriving from its navigation on the site be recorded.

2. answers are saved by clicking on the button “submit” and you could suspend the questionnaire at any time;

3. the sensitive personal data of all participants (including the IP address) are not tracked. The protocols on which information passes over the network and data is stored is the HTTPS (HyperText Transfer Protocol over SSL) protocol, a variant of the HTTP protocol that uses, in addition to TCP / IP, the SSL (Secure Sockets Layer) layer that encrypts incoming and outgoing data through a mathematical algorithm.”
