# Supplementary material for: Exploring the critical points of teaching STEM subjects in the time of COVID 19: the experience of the course "Microscopy Techniques for Forensic Biology"
Source: F1000Res. 2021 Apr 12;10:89. Originally published 2021 Feb 10. [Version 2] doi: 10.12688/f1000research.28455.2 (PMC8132475; doi:10.12688/f1000research.28455.2)
Supplement: Peer review history

# Peer review: Reviewer response for version 2

**DOI:** 10.5256/f1000research.55589.r83096

**Published:** 2021-05-18

**Authors:** Alexander H. Treusch

**Affiliations:** 1 Department of Biology, University of Southern Denmark, Odense, Denmark

I have to thank the authors for addressing all my comments and suggestions. Well done and I have no further comments.

Is the work clearly and accurately presented and does it cite the current literature?

Yes

If applicable, is the statistical analysis and its interpretation appropriate?

Yes

Are all the source data underlying the results available to ensure full reproducibility?

Partly

Is the study design appropriate and is the work technically sound?

Yes

Are the conclusions drawn adequately supported by the results?

Yes

Are sufficient details of methods and analysis provided to allow replication by others?

Partly

Reviewer Expertise:

Microbiology, environmental microbiology, implementation of virtual laboratory simulations into teaching.

I confirm that I have read this submission and believe that I have an appropriate level of expertise to confirm that it is of an acceptable scientific standard.

# Peer review: Reviewer response for version 2

**DOI:** 10.5256/f1000research.55589.r83257

**Published:** 2021-04-23

**Authors:** Maria Teresa Caccamo

**Affiliations:** 1 Department of Mathematical and Computer Sciences, Physical Sciences and Earth Sciences, Università di Messina, Messina, Italy

In the paper titled *“Exploring the critical points of teaching STEM subjects in the time of COVID 19: the experience of the course "Microscopy Techniques for Forensic Biology”, *the authors describe and analyze the online teaching experience carried out for the course of Microscopy Techniques for Forensic Biology offered as a part of the Master's degree program in Biology at the University of Calabria (Italy). In particular, the authors investigated the accessibility of distance learning along with an evaluation of adjustments needed for the conversion from offline to online instruction highlighting learning material and lesson duration, with specific emphasis on practical activities. From this new kind of approach emerged that the educational relationship between teacher and student has changed.

 The authors accurately explain how the data were collected and analyzed. The subject matter is within the scope of the journal and in terms of originality, subject coverage, clarity of presentation, strength of data and importance in field the paper is excellent. The article is novel and interesting to warrant indexing; furthermore, technically it adheres to the journal's standards. The title accurately reflects the content, the language is correct and the abstract is clear and adequate.

 I strongly encourage the indexing of this manuscript.

Is the work clearly and accurately presented and does it cite the current literature?

Yes

If applicable, is the statistical analysis and its interpretation appropriate?

Yes

Are all the source data underlying the results available to ensure full reproducibility?

Yes

Is the study design appropriate and is the work technically sound?

Yes

Are the conclusions drawn adequately supported by the results?

Yes

Are sufficient details of methods and analysis provided to allow replication by others?

Yes

Reviewer Expertise:

Physics, education, history of Physics

I confirm that I have read this submission and believe that I have an appropriate level of expertise to confirm that it is of an acceptable scientific standard.

# Peer review: Reviewer response for version 1

**DOI:** 10.5256/f1000research.31494.r79301

**Published:** 2021-03-17

**Authors:** Alexander H. Treusch

**Affiliations:** 1 Department of Biology, University of Southern Denmark, Odense, Denmark

The study “Exploring the critical points of teaching STEM subjects in the time of COVID 19: the experience of the course "Microscopy Techniques for Forensic Biology" by Brunelli and Macirella describes and analyzes the adaptation of a university course from in person to remote as a result of the lockdown under the COVID-19 pandemic.

 The study is an important contribution to the discussion on how we can bring the still fairly often traditional, offline university education in natural sciences into the e-learning age. Especially courses that include components designed to transfer practical skills are challenging in this regard, a topic that the study tries to address.

 While I think the research framework is well explained, it would be useful for a better understanding of the background of the students to include more information on the regular, pre-COVID course proceedings. Are the students usually using e-learning tools? If yes, which? Is there usually a digital exchange of learning materials (slides, assignments, etc.)? How much are instructional videos used? I would expect that students not used to e-learning tools at all will show a different reaction to those that have used them supporting an in-person course before and only are forced to use them exclusively now.

 Another thing that would be interesting to include (if the data is available) is how the learning goals were reached with moving the course to online teaching. I assume certain goals, especially of the hands-on training type, would be harder to reach in a remote learning setting. In this context, reporting the results of the evaluation (exam?) in comparison to previous years would give an indication of how well this was managed. However, only downstream courses that require the skills taught here and ultimately the students’ ability to fulfill their tasks in a future employment situation will determine if their learning success was affected by the remote teaching.

 While the authors mention virtual laboratory simulations in the discussion, I am wondering if there was a conscious decision made to not use them in the course and if yes, why? Simulations are usually considered very well suited for training students on instruments or letting them experience the workflow of a certain method/procedure. Therefore they are widely used in the training for the operation of technical equipment (e.g. planes, large machines), which are fairly similar, if not more complicated applications to the usage of microscopes.

 While, in general, I agree with the discussion and conclusions of the authors, I think there is another aspect to it that is not taken into consideration. Being unfamiliar with the Italian university education system, I get the feeling from the description the authors give in the manuscript that there are quite some differences to e.g. the system in Scandinavian countries. This could explain some of the results obtained by this study. For example, the observed promotion of communication by remote teaching and a subsequent build of trust between students and teachers furthering the academic discussions seems to be something coming from a completely different baseline than what I experience. This could indicate the possibility of a different teaching and learning culture in different countries and might be directly or indirectly related to the fact of how much the use of e-learning tools is already embraced. To be able to generalize the findings of this study I feel this is something that needs to be taken into consideration and discussed.

 Although I agree with the authors’ statement that e-learning tools have their limits in educations requiring the training of hands-on skills, there already is quite some progress with the emerging use of virtual laboratory simulations. However, similar to the authors I think they can only supplement the practical parts of a course and not completely replace them. Nevertheless, they are useful tools also outside of an emergency situation as what we have experienced the last year.

 Overall, I think this is a great contribution to the ongoing discussion on implementing e-learning tools into STEM educations. It could benefit from some additional clarifications to better put the study into perspective for people stemming from different university systems.

Is the work clearly and accurately presented and does it cite the current literature?

Yes

If applicable, is the statistical analysis and its interpretation appropriate?

Yes

Are all the source data underlying the results available to ensure full reproducibility?

Partly

Is the study design appropriate and is the work technically sound?

Yes

Are the conclusions drawn adequately supported by the results?

Yes

Are sufficient details of methods and analysis provided to allow replication by others?

Partly

Reviewer Expertise:

Microbiology, environmental microbiology, implementation of virtual laboratory simulations into teaching.

I confirm that I have read this submission and believe that I have an appropriate level of expertise to confirm that it is of an acceptable scientific standard, however I have significant reservations, as outlined above.

# Response

**Published:** 2021-03-19

**Authors:** Elvira Brunelli

**Affiliations:** University of Calabria, Italy

The study “Exploring the critical points of teaching STEM subjects in the time of COVID 19: the experience of the course "Microscopy Techniques for Forensic Biology" by Brunelli and Macirella describes and analyzes the adaptation of a university course from in person to remote as a result of the lockdown under the COVID-19 pandemic.

 The study is an important contribution to the discussion on how we can bring the still fairly often traditional, offline university education in natural sciences into the e-learning age. Especially courses that include components designed to transfer practical skills are challenging in this regard, a topic that the study tries to address.

 While I think the research framework is well explained, it would be useful for a better understanding of the background of the students to include more information on the regular, pre-COVID course proceedings. Are the students usually using e-learning tools? If yes, which? Is there usually a digital exchange of learning materials (slides, assignments, etc.)? How much are instructional videos used? I would expect that students not used to e-learning tools at all will show a different reaction to those that have used them supporting an in-person course before and only are forced to use them exclusively now.

**Thank you for the suggestions. I accepted and I inserted in the Introduction section a sentence to better clarify the background of students.**

****

**Despite the good level of confidence with digital tools (developed in different, non-educational contexts), students used few digital resources during the regular pre-COVID courses. In general, the slides showed during the lessons are provided, but both the books and the assignments are of traditional type; moreover, instructional videos are not employed, and the laboratory experiences are delivered during practical laboratory sessions.**

 Another thing that would be interesting to include (if the data is available) is how the learning goals were reached with moving the course to online teaching. I assume certain goals, especially of the hands-on training type, would be harder to reach in a remote learning setting. In this context, reporting the results of the evaluation (exam?) in comparison to previous years would give an indication of how well this was managed. However, only downstream courses that require the skills taught here and ultimately the students’ ability to fulfill their tasks in a future employment situation will determine if their learning success was affected by the remote teaching.

 For what concerns the examination, the outcome was not different from that of previous cohorts, and all students passed the exams in the ordinary academic session. Instead, I noticed an increase in the internship request for the preparation of the final dissertation. I did not include these data in the paper because it would be better clarified if it was a stochastic event or determined by a curiosity about microscopy developed during the course and not entirely satisfied.

**I agree that the practical consequences of such online experiences on student formation are delayed, and we cannot at the moment establish if all the students acquired the appropriate practical microscopic skill.**

 While the authors mention virtual laboratory simulations in the discussion, I am wondering if there was a conscious decision made to not use them in the course and if yes, why? Simulations are usually considered very well suited for training students on instruments or letting them experience the workflow of a certain method/procedure. Therefore they are widely used in the training for the operation of technical equipment (e.g. planes, large machines), which are fairly similar, if not more complicated applications to the usage of microscopes.

**During the course last year, I did not use virtual laboratory since we were absolutely unprepared: the course started in March together with the first lockdown. However, thanks to such experience, I am using a virtual laboratory now, since in Italy we are still in a serious pandemic situation and lessons are mainly delivered online. I hope to merge both methods this year since I am convinced of the utility of simulations to make the student mindful about the methods applied and skills required.**

 While, in general, I agree with the discussion and conclusions of the authors, I think there is another aspect to it that is not taken into consideration. Being unfamiliar with the Italian university education system, I get the feeling from the description the authors give in the manuscript that there are quite some differences to e.g. the system in Scandinavian countries. This could explain some of the results obtained by this study. For example, the observed promotion of communication by remote teaching and a subsequent build of trust between students and teachers furthering the academic discussions seems to be something coming from a completely different baseline than what I experience. This could indicate the possibility of a different teaching and learning culture in different countries and might be directly or indirectly related to the fact of how much the use of e-learning tools is already embraced. To be able to generalize the findings of this study I feel this is something that needs to be taken into consideration and discussed.

**I agree. This would be an interesting topic to be investigated. I inserted in the text some sentences to introduce these concepts (highlighted). I also suggest that emotional reaction to the worrying pandemic situation may have been relevant in determining the students' reaction. However, this is out of my competencies and expertise.**

 Although I agree with the authors’ statement that e-learning tools have their limits in educations requiring the training of hands-on skills, there already is quite some progress with the emerging use of virtual laboratory simulations. However, similar to the authors I think they can only supplement the practical parts of a course and not completely replace them. Nevertheless, they are useful tools also outside of an emergency situation as what we have experienced the last year.

 Overall, I think this is a great contribution to the ongoing discussion on implementing e-learning tools into STEM educations. It could benefit from some additional clarifications to better put the study into perspective for people stemming from different university systems.
